# AI-Powered Mice Behavior Tracking and Its Application for Neuronal Manifold Analysis Based on Hippocampal Ensemble Activity in an Alzheimer’s Disease Mice Model

**DOI:** 10.3390/ijms26157180

**Published:** 2025-07-25

**Authors:** Evgenii Gerasimov, Viacheslav Karasev, Sergey Umnov, Viacheslav Chukanov, Ekaterina Pchitskaya

**Affiliations:** 1 Laboratory of Molecular Neurodegeneration, Peter the Great St. Petersburg Polytechnic University, St. Petersburg 194021, Russia; 2 Laboratory of Biomedical Imaging and Data Analysis, Peter the Great St. Petersburg Polytechnic University, St. Petersburg 194021, Russia; 3 Laboratory of Molecular Neurobiology, Pavlov Institute of Physiology, Russian Academy of Sciences, St. Petersburg 199034, Russia

**Keywords:** neuronal activity, manifold, behavioral classification, mice tracking, YOLO, neuronal network, artificial intelligence, calcium imaging, Alzheimer’s disease, locomotion

## Abstract

Investigating brain area functions requires advanced technologies, but meaningful insights depend on correlating neural signals with behavior. Traditional mice behavior annotation methods, including manual and semi-automated approaches, are limited by subjectivity and time constraints. To overcome these limitations, our study employs the YOLO neural network for precise mice tracking and composite RGB frames for behavioral scoring. Our model, trained on over 10,000 frames, accurately classifies sitting, running, and grooming behaviors. Additionally, we provide statistical metrics and data visualization tools. We further combined AI-powered behavior labeling to examine hippocampal neuronal activity using fluorescence microscopy. To analyze neuronal circuit dynamics, we utilized a manifold analysis approach, revealing distinct functional patterns corresponding to transgenic 5xFAD Alzheimer’s model mice. This open-source software enhances the accuracy and efficiency of behavioral and neural data interpretation, advancing neuroscience research.

## 1. Introduction

In modern neuroscience, understanding neuronal ensemble activity and connectivity is one of the most urgent challenges. Investigating how different brain regions function in vivo requires advanced technologies, such as electrode arrays [1,2,3], microendoscopes [4,5,6,7,8], and two-photon imaging [9,10,11]. However, gaining meaningful information from these neural signals necessitates their correlation with the animal’s behavior [12]. This validation process has traditionally relied on various approaches, including manual identification of an animal’s position and behavior type [13,14], semi-automated techniques [15,16,17], and AI-driven methods [12,18,19]. Manual behavior annotation is inherently limited by investigator-dependent accuracy, introducing potential biases in data interpretation. Different researchers may classify identical behaviors differently, leading to inconsistencies [20]. Additionally, manual scoring is an extremely time-consuming process, often requiring several hours to annotate just a few minutes of behavioral data. These limitations emphasize the need for a highly efficient and automated approach to accurately determine animal behavior in neuroscience research.

To address this challenge, our study leverages the YOLO-Pose neural network for precise mice tracking [21] and behavioral scoring, utilizing reference points based on the MARS system [12]. Our findings indicate that the YOLO-based model effectively and accurately identifies key reference points on a mouse’s body. For further behavioral validation, we incorporated a composite RGB frames method, ensuring robust and reliable classification of movement patterns [22]. The YOLO neural network was trained on a dataset comprising more than 10,000 annotated frames across three primary behaviors: sitting, running, and grooming. In addition to behavior tracking, our open-source software package provides comprehensive statistical metrics, including average speed, time spent in specific zones, ethograms, and data visualization tools. To further demonstrate the reliability and applicability of our approach, we utilized AI-powered behavior labeling to investigate hippocampal neuronal ensemble activity recorded in vivo using miniature fluorescence microscopy [23]. This technique allows for simultaneous monitoring of hundreds of simultaneously active neurons in freely behaving mice. Correlating corresponding behavioral states with neuronal activity might provide deeper insights into brain function.

To analyze the intricate dynamics of neuronal circuits, we implemented a neuronal manifold analysis approach [24,25,26]. This method, based on dimensionality reduction techniques, was applied to preprocessed calcium imaging data [27,28], enabling simultaneous assessment of all recorded neuronal calcium signals within biologically relevant timeframes. Given the complex organization of brain neuronal circuits, where neuronal activity can exhibit significant variability across individual neurons and experimental conditions, manifold analysis provides a powerful tool for uncovering distinct patterns in brain region functionality [24]. To validate both AI-based mice tracking and behavior classification alongside neuronal manifold analysis, we conducted experiments on wild-type and 5xFAD transgenic mice, a well-established model of Alzheimer’s disease [29,30,31,32,33]. The 5xFAD mice exhibit aberrant hippocampal neuron activity and severe cognitive impairments, making them a suitable model for evaluating the presented approach [34,35]. Our results revealed abnormal neuronal manifold compositions in transgenic mice both in total representation and during distinct behavior-related epochs.

In summary, this paper presents a powerful open-source software package designed for automated animal behavior analysis and high-order neuronal activity examination. By integrating AI-based behavioral tracking with advanced neuronal activity analysis, this tool significantly enhances the accuracy, efficiency, and depth of behavioral and neural data interpretation in neuroscience research.

## 2. Results

### 2.1. Artificial Intelligence-Powered Mice Tracking Using Pretrained YOLO-Pose-v8

**Figure 1:**
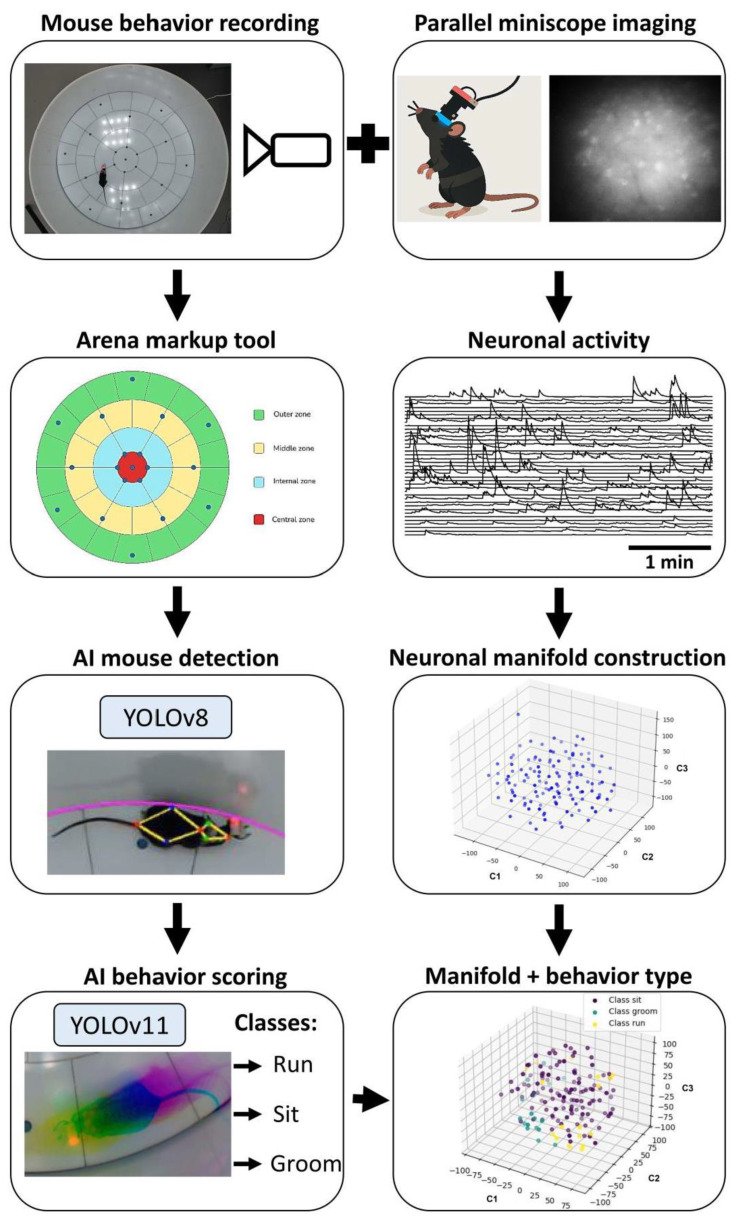
Schematic illustration of the experimental pipeline for AI-driven mouse position estimation and behavior scoring, integrated with neuronal manifold construction based on hippocampal activity recorded using a miniscope.

Mice position tracking is essential for validating its spatial location during different behavioral tests. Moreover, various crucial behavior describing parameters are also tightly bound to an accurate and efficient mice tracking and behavioral type estimation during different tasks [36,37,38,39,40,41,42]. Furthermore, combining neuronal activity recordings with behavioral data in freely moving mice can provide essential insights into brain region functioning. To investigate this, we employed artificial intelligence-based methods for mouse tracking and behavior scoring, integrated with neuronal manifold constraints. An overall pipeline is presented in Figure 1.

**Figure 2:**
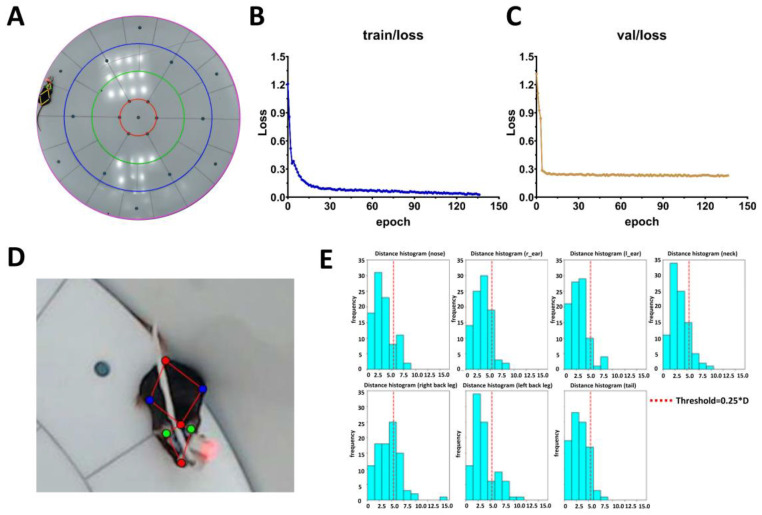
Mice tracking and core point estimation using the pretrained YOLO-Pose-v8 neuronal network. (**A**) Tracking mice position in the rounded arena using pretrained YOLO-Pose-v8. Red line is a boarder of the center zone, green line is a boarder of middle zone, blue line is a boarder of outer zone and pink line is a boarder of edge zone. (**B**) Loss curve of YOLO-Pose-v8 during training epochs. (**C**) Loss curve of YOLO-Pose-v8 during validation epochs. (**D**) Mouse in the rounded arena with highlighted core points by the MARS system. (**E**) Histogram distribution of error distances from manual mice core-point annotations. A red dotted line threshold of 0.25*D (PCK@0.25) is presented.

For automatic estimation of mice positioning, we have leveraged neuronal network (NN) of YOLO-Pose-v8 [43]. The network was pretrained to validate mice position via core points on the mice body highlighted by the MARS system [12]. The neuronal network was trained for estimation of mice positioning in the rounded arena (Figure 2A). The total manually marked up dataset by the MARS system consisted of 500 frames of freely behaving mouse in the rounded arena. Rounded arena zone validation was established using computer vision methods and is described in detail in the Section 4 and Supplementary Figure S1.

For YOLO-Pose-v8 training, we used 375 pre-marked frames of a behavioral recording video with a 1920 × 1080 resolution. Learning curves for training epochs are presented in Figure 2B. As a validation dataset, 175 frames with mouse behavior that have never been presented to a neuronal network model were used. Validation curves are shown in Figure 2C.

To validate the quality of core-point tracking percentage of correct key points (PCK) metric was applied [44]. In this metric, a constant D (in this case, D = 0.25) is used to multiply the distance between the right and left mouse ears. The resulting value represents a confidence radius. If the predicted point lies within a circle with a radius equal to the confidence radius and centered on the true point, we can conclude that the neural network has successfully marked a distinct point (Figure 2D,E).

However, the lowest percentage of correct point detection was observed for both hind legs. This is likely due to the high movement of these body parts. The mean PCK metric value was 81.9% ± 3.3%. The distribution of marking errors relative to the manually annotated points is shown in Figure 2E. A red dotted line represents the pixel threshold (0.25 × D = 5.2 pixels), with most of the NN-marked points positioned to the left of this threshold. To compare our results with publicly available AI-based approaches, we trained DeepLabCut [18] on the same dataset—375 manually pre-annotated training frames and 125 validation frames. DeepLabCut allows researchers to track animal behavior by marking four key points: the nose, left ear, right ear, and tail. For evaluation, we used the PCK metric with the same D = 0.25 parameter, and the results are summarized in Table 1. For visual comparison of neuronal networks performance, one can find videos with pretrained YOLO-Pose-v8 and DeepLabCut in Video S1.

Our approach, based on the pretrained YOLO-Pose-v8 neural network, demonstrated significantly higher performance on the small training dataset compared to DeepLabCut. However, DeepLabCut may require a much larger training dataset for optimal performance in tracking mice. In contrast, our method maintained stable key-point validation and tracking performance, making it more efficient, particularly on relatively small training datasets.

### 2.2. Artificial Intelligence-Powered Mice Behavior Scoring Using Pretrained YOLO-Pose-v11

**Figure 3:**
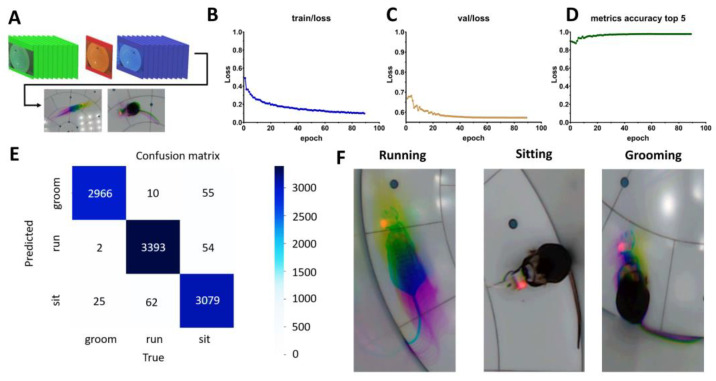
Determination of mice behavior type with pretrained YOLO-Pose-v11. (**A**) Composite frames approach for mice behavior scoring. (**B**) Loss curve of YOLO-Pose-v11 during training epochs. (**C**) Loss curve of YOLO-Pose-v11 during validation epochs for parameters fitting. (**D**) Accuracy of pretrained YOLO-Pose-v11 at identification mice behavior type. (**E**) Confusion matrix of pretrained YOLO-Pose-v11 at estimation of behavior. (**F**) Illustrative composite frames composition for distinct types of behavior as running, sitting and grooming, respectively.

To move from mouse position tracking towards behavioral scoring, we trained the next generation of the YOLO-Pose-v11 neuronal network, which incorporates an attention mechanism in its architecture [45]. This model was designed to distinguish between three distinct types of behavior: running, sitting, and grooming. Unlike other characteristics of mice, behavior is not determined by a single frame but by a sequence of frames, as actions unfold over time. Therefore, attempting to extract behavior from individual frames would be ineffective. To address this, a specialized data format—composite frames—was developed for training [22]. The concept is as follows: a sequence of 21 frames is captured, with behavior analyzed in the 11th frame. The first 10 frames are processed by extracting, summing, and averaging their green channels (Figure 3A). The 11th frame retains only the red channel. The last 10 frames are processed by extracting, summing, and averaging their blue channels. These three channels are then combined into a single RGB composite image (Figure 3A, bottom).

To construct an accurate classifier, it was essential to identify the various types of activities that occur on composite frames. For this purpose, YOLO-Pose-v11 was pretrained on more than 28,900 frames with 90 learning epochs. The training curve is presented on Figure 3B. Further, model parameters were fine-tuned on the validation dataset (Figure 3C). To evaluate the effectiveness of the pretrained YOLO-Pose-v11, we assessed its consistency in behavioral scoring across 9646 frames of mouse behavior. The model’s accuracy was defined as the probability of correctly classifying a behavior based on manual annotations (Figure 3D). After 25 testing epochs, accuracy ranged between 0.96 and 0.98, and the F-score reached 0.971. The confusion matrix (Figure 3E), demonstrates highly accurate classification across behavior categories, each of which is illustrated in Figure 3F.

However, distinguishing grooming from sitting remains a challenge due to the similarity between these behaviors and the variability of grooming actions. The problem is exacerbated by the single camera angle, which makes it difficult even for human observers to determine mouse behavior accurately. Additionally, the neural network occasionally misclassified behaviors, resulting in sequences where some frames are incorrectly categorized. To mitigate these issues, a two-stage data filtering process was implemented, starting with Kalman filtering [46]. A key advantage of this approach is the “behavior matrix,” which redistributes the probability of observing different behaviors, reducing classification errors caused by camera noise and other disturbances. Further, smoothing by the mode was performed. In this approach, a series of 25 frames were analyzed, and the behavior label for all frames in the sequence is determined by the most frequently occurring behavior.

**Figure 4:**
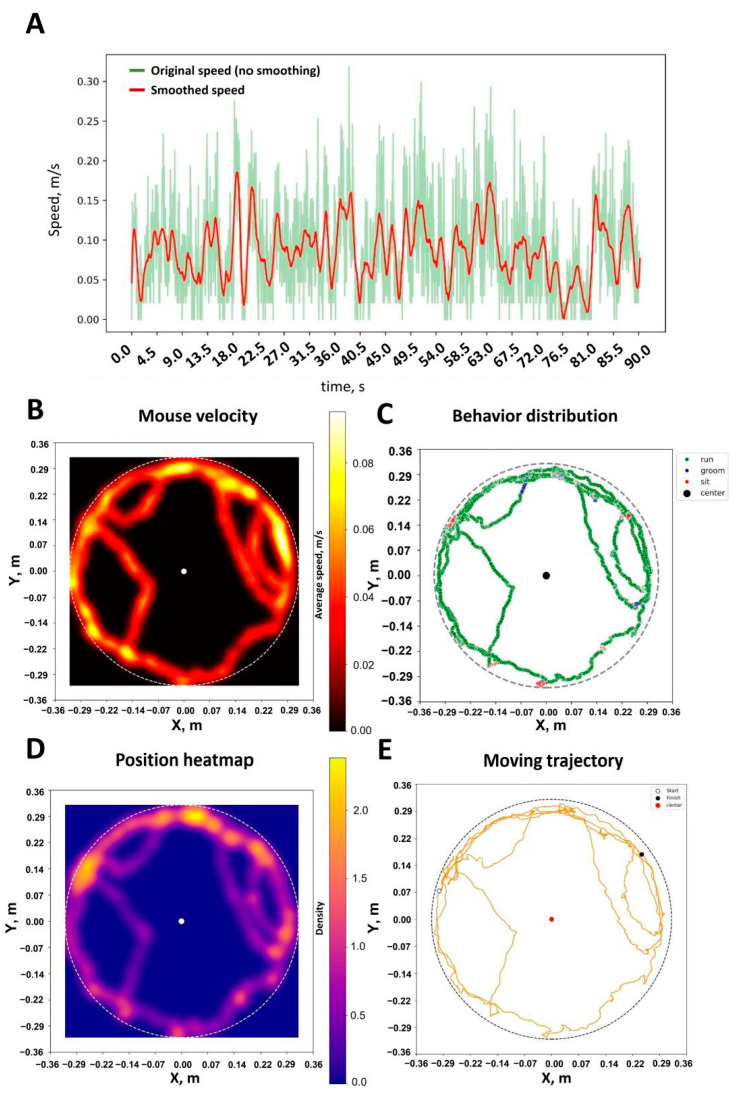
Visualization of the mice movement and behavioral scoring results. (**A**) Mouse speed visualization for each frame—original (green line) and smoothed (red line). (**B**) Mouse velocity heatmap. (**C**) Movement distribution when freely moving in the rounded arena. (**D**) Position heatmap of mouse. (**E**) Mouse moving trajectory. All graphs illustrated single mouse behavior in the rounded arena.

After mouse tracking and behavior classification via the pretrained neuronal network, all data are saved in the user-friendly “.csv” format so users might work with them for further analysis. At the same time, all the results describing mice behavior are easily visualized (Figure 4). Researchers could find a raw and smoothed mouse velocity (Figure 4A) and its distribution heatmap (Figure 4B). Results based on the behavior classification (Figure 4C), position heatmap (Figure 4D) and moving trajectory (Figure 4E) could also be visualized and saved for the following analysis.

### 2.3. Neuronal Manifold Construction in Normal and Pathological Conditions Based on Miniature Fluorescence Calcium Imaging

Modern techniques for recording neuronal ensemble activity, such as miniscopes and microendoscopes, provide researchers with a vast amount of information regarding neuronal cell function and connectivity across various brain regions [4,6,8,34,47,48,49]. These methods rely on calcium imaging, an indirect proxy for neuronal activity [9,50,51,52]. In this study, we utilized the genetically encoded calcium indicator GCaMP6f to visualize neuronal activity [53]. The fluorescence intensity of GCaMP6f increases rapidly in response to neuronal activation [54]. We recorded the activity of dorsal hippocampal neurons in freely behaving mice within a rounded arena. Imaging sessions, each lasting five minutes, were conducted once per day over four consecutive days. To examine the composition and structure of neuronal manifolds in a normal state, wild-type mice (WT+veh) were used as controls. To assess pathological changes in the hippocampal activity during free movement, we employed the 5xFAD mouse model of Alzheimer’s disease (5xFAD+veh). Additionally, we investigated the efficacy of neuronal manifold analysis based on miniscope data by conducting calcium imaging in a treated 5xFAD mouse group (5xFAD+treat). All mice used in the experiments were 6.5 months old, as this is the age at which pronounced pathological changes typically emerge in 5xFAD transgenic mice [32,55].

**Figure 5:**
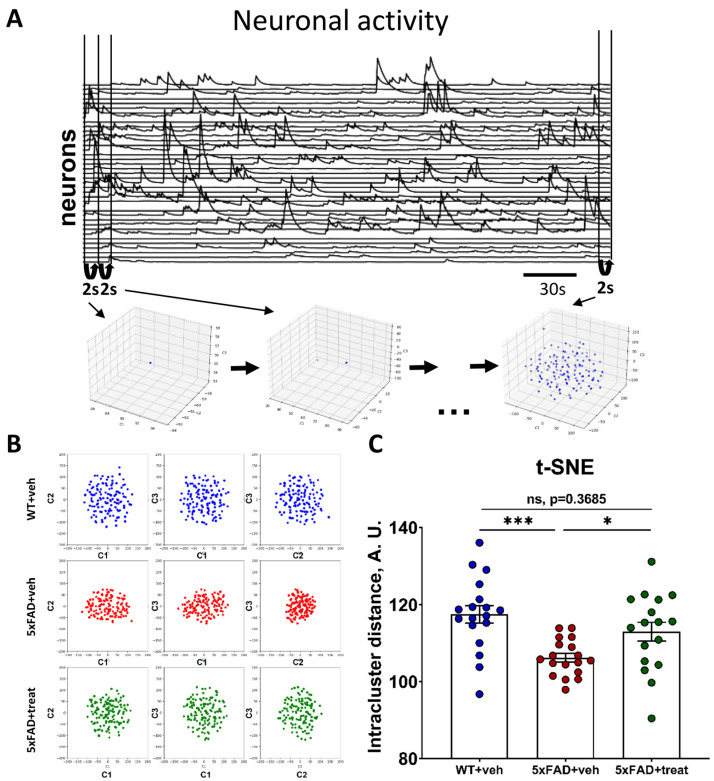
Neuronal manifolds constrained on the miniature fluorescence microscopy data. (**A**) Schematic illustration of the neuronal manifold construction of the single recording based on the neuronal calcium traces. (**B**) Two-dimensional representation of the neuronal manifold architecture for WT+veh, 5xFAD+veh and 5xFAD+treat mice groups. (**C**) Significant decrease in the neuronal manifold intracluster distance is observed in the 5xFAD mice control group. WT+veh: n = 18 sessions, N = 7 mice; 5xFAD+veh: n = 18 sessions, N = 6 mice; and 5xFAD+treat: n = 17 sessions, N = 6 mice. Brown–Forsythe and Welch–ANOVA following Games–Howell’s multiple comparisons test was used (ns: non-significant, *: *p* < 0.05, ***: *p* < 0.001). All data are presented as the mean ± SEM.

Preprocessing of the acquired data was performed using the publicly available Minian pipeline, which facilitated background estimation, motion correction, and neuronal calcium trace extraction [27,56]. Based on the obtained fluorescence intensities for each neuron, we leveraged dimensionality reduction (DR) methods for constraining neuronal manifolds [57,58]. For analysis, each five-minute recording was segmented into two-second intervals, with DR performed for each segment (Figure 5A). We evaluated both linear (ISA, MDS, and PCA) and nonlinear (UMAP and t-SNE) DR techniques to determine their ability to distinguish between the different mouse groups based on neuronal manifold architecture (Figure S2). The t-stochastic neighbor embedding (t-SNE) method yielded the most consistent results (Figure 5B). The effectiveness of these methods was assessed using the intracluster distance metric (Figure 5C).

The mean intracluster distances of neuronal manifolds in control transgenic 5xFAD mice were significantly reduced compared to their wild-type littermates (WT+veh: 117.5 ± 2.2 vs. 5xFAD+veh: 106.2 ± 1.1, *p* = 0.0004) and were lower than those in the treated 5xFAD group (5xFAD+veh: 106.2 ± 1.1 vs. 5xFAD+treat: 112.9 ± 2.4, *p* = 0.0489). However, the treated 5xFAD mice exhibited neuronal manifold structures comparable to WT+veh mice, as reflected in the absence of significant differences in intracluster distances (WT+veh: 117.5 ± 2.2 vs. 5xFAD+treat: 112.9 ± 2.4, *p* = 0.3685, Brown–Forsythe and Welch–ANOVA tests following Games–Howell’s multiple comparisons post hoc test) (Figure 5C). Other DR methods, such as ISA, were not powerful enough, and some failed to segregate the mice groups altogether (Supplementary Figure S2).

To further validate the reliability of intracluster distance as a descriptor of neuronal manifold structure, we trained a linear encoder to classify mice based on intracluster distance values (Table 2). This threshold-based classifier, using intracluster distance as a single feature, effectively distinguished 5xFAD+veh mice from both other groups. To confirm that classification was based on biologically meaningful temporal coordination rather than random correlations, we conducted the analysis on phase-randomized calcium imaging data (Table 2).

Shuffling eliminated the classifier’s discriminative power, reducing accuracy to chance levels. This null result confirms that the observed group differences stem from biologically meaningful temporal coordination within the hippocampal ensembles of the mice hippocampus. Furthermore, the clear classification of both WT+veh and 5xFAD+treat mice, distinct from the 5xFAD+veh group, highlights the highly aberrant and disrupted neuronal activity in the hippocampus of 5xFAD control mice, leading to an altered neuronal manifold structure. Notably, hippocampal neuronal circuits in the 5xFAD+treat group exhibited characteristics of beneficial treatment effects, as reflected in the increased intracluster distance (Figure 5C) and the reduced ability of the encoder to distinguish between WT+veh and 5xFAD+treat mice (Table 2).

### 2.4. Altered Neuronal Manifold Composition in the Transgenic 5xFAD Mice During Different Behavioral Types

**Figure 6:**
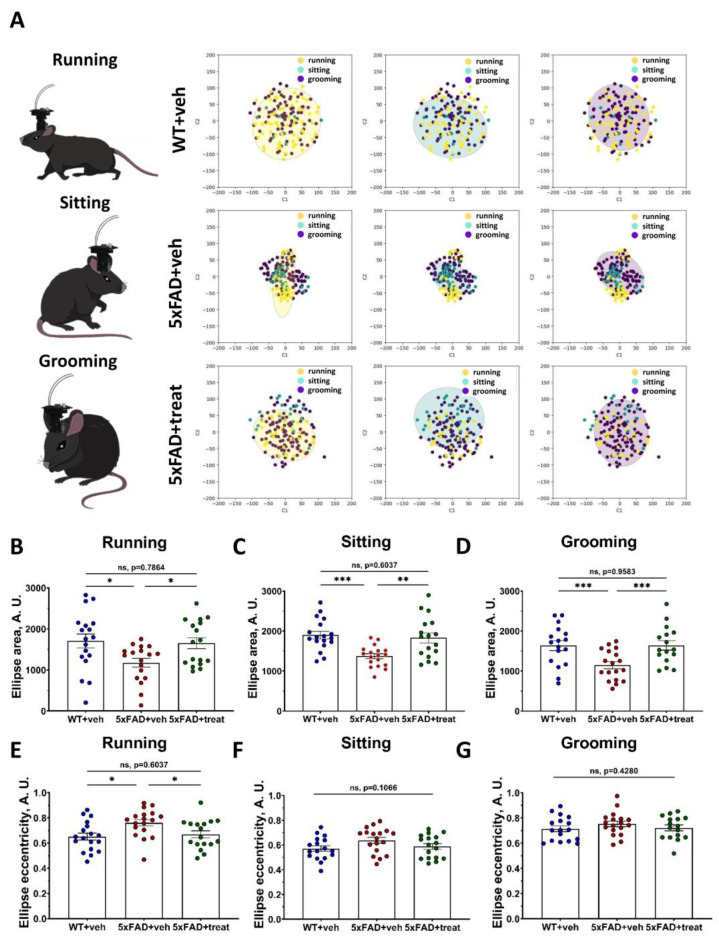
Neuronal manifold geometrical features belonging to distinct behavior types are highly aberrant in transgenic 5xFAD mice. (**A**) Ellipses fitting for description of neuronal manifold architecture for running, sitting and grooming states. (**B**–**D**) Ellipse area constrained on the neuronal manifolds for running, sitting and grooming epochs, respectively. (**E**–**G**) Ellipses eccentricity based on the neuronal manifolds for running, sitting and grooming types of behavior, respectively. WT+veh: n = 18 sessions, N = 7 mice; 5xFAD+veh: n = 18 sessions, N = 6 mice and 5xFAD+treat: n = 17 sessions, N = 6 mice. Ordinary one-way ANOVA following Holm–Sidak’s post hoc test was used (*: *p* < 0.05, **: *p* < 0.01, ***: *p* < 0.001). All data are presented as the mean ± SEM.

Altered hippocampal neuron functioning in Alzheimer’s disease mouse models is well established [59,60,61,62]. The accumulation of toxic Aβ-amyloid plaques plays a critical role in disrupting neuronal excitability and impairing neuron-to-neuron communication [32,63,64], primarily due to the extensive loss of spine apparatus function [65,66,67,68]. In the previous section, we demonstrated these functional alterations in the dorsal hippocampus using neuronal manifold analysis and encoder classification based on intracluster distances. However, it remains unclear whether neuronal activity associated with distinct behavioral states is similarly abnormal in transgenic 5xFAD mice [69]. To address this question, we utilized neuronal manifold representations to analyze hippocampal ensemble activity during specific behavioral states, including running, sitting, and grooming (Figure 6A). Behavior scoring was performed using the AI-powered approach for mouse tracking and behavior classification described earlier. For each two-second interval, we identified the predominant behavior and mapped each point within the neuronal manifold to its corresponding behavioral state (Figure 6A). To characterize the complex structure of the neuronal manifolds, we fitted ellipses to each manifold projection and compared their geometric properties, including eccentricity and covered area (Figure 6B–G).

Using geometrical features of ellipses, we described the properties of neuronal manifolds. This approach allowed us to identify alterations in the hippocampal neuronal circuits of freely moving transgenic 5xFAD mice during distinct behavioral states. Specifically, we analyzed the area of ellipses derived from neuronal manifolds to investigate global changes in hippocampal neuronal functioning. Across all classified behavioral states—running, sitting, and grooming (Figure 6A), the ellipses’ areas were significantly altered in 5xFAD control mice compared to their wild-type littermates (running: WT+veh: 1706 ± 169 vs. 5xFAD+veh: 1176 ± 106, *p* = 0.0258; sitting: WT+veh: 1901 ± 94 vs. 5xFAD+veh: 1371 ± 61, *p* = 0.0007; grooming: WT+veh: 1635 ± 123 vs. 5xFAD+veh: 1145 ± 88, *p* = 0.0071) (Figure 6B–D). Treatment of 5xFAD mice led to a significant restoration of these values towards wild-type levels (running: 5xFAD+veh: 1176 ± 106 vs. 5xFAD+treat: 1652 ± 132, *p* = 0.0380; sitting: 5xFAD+veh: 1176 ± 106 vs. 5xFAD+treat: 1830 ± 125, *p* = 0.0028; grooming: 5xFAD+veh: 1176 ± 106 vs. 5xFAD+veh: 1643 ± 118, *p* = 0.0071, for all comparisons; ordinary One-way ANOVA following Holm–Sidak’s post hoc test were applied) (Figure 6B–D). Notably, treatment restored ellipse areas to levels from those of wild-type mice (*p* > 0.6037) for all types of behavior.

Next, we assessed the eccentricity of the ellipses (Figure 6E–G). Significant differences in eccentricity were observed only during running behavior, where 5xFAD+veh mice exhibited markedly altered manifold architecture compared to both WT+veh and 5xFAD+treat groups (WT+veh: 0.65 ± 0.03 vs. 5xFAD+veh: 0.76 ± 0.03, *p* = 0.018; 5xFAD+veh: 0.76 ± 0.03 vs. 5xFAD+treat: 0.67 ± 0.028, *p* = 0.0440) (Figure 6E–G). To determine whether these geometric features could be used to classify behavioral states, we trained an encoder based on the neuronal manifolds’ geometric descriptor (Table 3).

To validate the specificity of this approach, neuronal activity was shuffled, which—as shown in the previous chapter—significantly impaired the encoder’s classification performance, confirming that the observed results were not due to random patterns. Furthermore, the encoder was able to clearly distinguish WT+veh and 5xFAD+treat mice from the 5xFAD+veh group, underscoring the presence of aberrant and disrupted hippocampal activity in the control transgenic mice. Importantly, the restoration of neuronal ensemble dynamics across all behavioral states in the 5xFAD+treat group was reflected in both the normalization of ellipse descriptor (Figure 6B–G) and the diminished ability of the encoder to differentiate between WT+veh and 5xFAD+treat animals (Table 3).

## 3. Discussion

Accurate and unbiased classification of mouse behavior is essential for understanding the functions of distinct brain regions, especially when neuronal activity is recorded simultaneously with behavioral observation [70,71,72,73,74]. Modern in vivo techniques such as multi-electrode arrays [75,76], miniscopes [6,77], and two-photon or multi-photon imaging through optical fibers [9,10] allow researchers to study how neuronal ensembles underpin behavior. Additionally, the development of genetically encoded calcium or voltage indicators has greatly expanded the ability to investigate specific neuronal subtypes across different brain areas [53,78,79]. However, while imaging technologies and genetically encoded tools have rapidly advanced, methods for comprehensive, high-throughput behavioral analysis have not kept pace. To address this gap, we introduce a novel AI-based approach for mouse tracking and behavioral scoring. Using the YOLO neural network architecture, we achieved highly accurate mice tracking through core-point estimation during free movement [21,43]. A small, manually annotated dataset—375 frames labeled using the MARS system—was sufficient to train the network effectively, resulting in robust tracking performance [12]. Compared to the widely used DeepLabCut [18], our method demonstrated superior accuracy and performance despite relying on a significantly smaller training dataset. A possible explanation for the better performance of the YOLO neural network in the task of mouse key-point annotation trained on small datasets, compared to DeepLabCut, lies in its architectural design. Specifically, the use of a focal loss function and additional feature extraction separating layers from the final prediction head likely contribute to improved generalization and accuracy [80]. It is also important to acknowledge a limitation of this comparison: for optimal performance in tracking mice, DeepLabCut typically requires a substantially larger training dataset, which may have affected its relative performance under the current experimental conditions.

While simple tracking is valuable, it is often insufficient for advanced neuroscience research, such as exploring brain function or evaluating therapeutic strategies for neurological disorders. More sophisticated behavior analysis is needed—one that can detect changes in behavioral states, spatial distributions, and transitions between behaviors [81,82]. To meet these needs, we utilized the YOLO-Pose-v11 network for automatic, unbiased classification of behavior types [45]. This latest YOLO generation includes attention mechanisms that enhance the robustness of the model. Training was conducted using over 28,900 composite frames with manually labeled behaviors, while fine-tuning was performed on an additional 9646 frames. After 25 training epochs, validation accuracy ranged from 0.94 to 0.96, with an F-score of 0.971—demonstrating the model’s ability to distinguish between different behavioral types. The output also supports visualization of spatial metrics such as distance traveled, mice velocity, zone occupancy, and behavior-specific heatmaps. It is important to note that both the type and amount of observable behavior are closely tied to the specific behavioral testing paradigm and experimental conditions employed. For instance, in learning and memory assessments—such as various versions of the Fear Conditioning (FC) test—freezing behavior is of primary interest, as it serves as a key indicator of context or associative memory [39]. At the same time, in behavior tests designed to investigate emotional changes or stress-related behaviors, such as anxiety or fear, a broader spectrum of behaviors becomes relevant [83]. These may include fleeing, freezing, and rapid locomotion (e.g., escape-like running), each offering unique insights into the emotional and physiological state of the animal. Given the diversity of behaviors relevant across different paradigms, the pretrained YOLO-Pose-v11 neuronal network, presented in the current article, could be further trained to recognize specific types of behavior as required by different testing paradigms.

To validate the presented AI-based approach, we applied it to study hippocampal neuronal activity in pathological conditions using the 5xFAD mouse model of Alzheimer’s disease [29,32]. The hippocampus is severely suffering in Alzheimer’s, showing ubiquitous amyloid plaque accumulation [84], disrupted calcium signaling [85,86], and dendritic spine degradation [66,87]. Neuronal activity was recorded using a miniscope, enabling imaging in freely moving mice. We further analyzed neuronal population dynamics using the neuronal manifold approach, which captures ensemble activity during defined intervals [26]. Among dimensionality reduction methods, t-SNE provided the best separation between groups, particularly distinguishing WT+veh and 5xFAD+vehicle mice based on intracluster distances. The 5xFAD+veh group showed significantly reduced intracluster distances, while the 5xFAD+treat group aligned more closely with WT animals. Next, we constructed behavioral state-specific manifolds (e.g., running, sitting, and grooming) and analyzed their geometric features. Alzheimer’s disease (AD) disrupts a wide range of physiological brain functions [88,89]. As a result, cognitive decline and memory impairment are hallmark features of AD. Beyond these cognitive deficits, previous studies have shown that hippocampal neurons in AD mouse models, such as the 3xTg-AD mice, exhibit significantly altered neuronal Ca^2+^ activity in response to locomotion [69]. This highlights the importance of investigating not only single-neuron activity but also the dynamics of broader hippocampal neuronal circuits under behaviorally relevant conditions [4,90]. In the present study, we introduce a three-class behavioral classification system—running, sitting, and grooming—based on mouse behavior in a rounded arena. These behaviors are particularly suitable for probing locomotion-associated neuronal circuit dysfunctions in the 5xFAD transgenic mouse model of Alzheimer’s disease. Ellipse fitting to manifold projections revealed significantly reduced area in the 5xFAD+veh group across all behaviors, with no differences between WT+veh and 5xFAD+treat groups.

Several hypotheses can be proposed to explain the observed findings. First, the significant reduction in intracluster distances in the 5xFAD+veh group may reflect abnormal functioning of hippocampal neurons. In these animals, most of the neuronal manifold data points lie close to each other, indicating a low variability in overall circuit activity. This reduced variability may be associated with neuronal hyperexcitability—a hallmark of AD pathology—as previously shown by our group in this dataset [90] and supported by other studies [91,92,93]. Importantly, intracluster distance analysis also highlights the beneficial effect of chronic intraperitoneal administration of NDC-9009 (10 mg/kg) in the 5xFAD+treat group. In these mice, the neuronal manifold architecture reverted to levels observed in WT+veh animals, suggesting a restoration of healthy network dynamics. This result strongly supports the use of neuronal manifold analysis as a systems-level approach to evaluate circuit-level dysfunction in neuropathological conditions. When correlating the neuronal manifold metrics with behavioral data, further specific changes in geometric features—particularly ellipse area—were observed. The 5xFAD+veh group showed a marked reduction in ellipse area, which may reflect aberrant coordination among hippocampal neurons in transgenic control animals [94]. These manifold characteristics align with our previous findings on 5xFAD mice treated with NDC-9009 [90] obtained via canonical quantitative analysis [28], thus validating the consistency and efficiency of the manifold approach. In this case, 5xFAD+treat mice group plays and essential role for comparisons and right results interpretation. Furthermore, the present findings are in agreement with prior single-neuron level analyses, where locomotion in 5xFAD mice elicited exaggerated neuronal responses [69]. Overall, manifold-constrained analysis of neuronal ensembles offers a powerful method to assess large-scale network dynamics. Unlike analyses focused solely on individual neuronal activity or pairwise connectivity, the manifold approach enables an integral view of circuit behavior [58], potentially uncovering pathological patterns that would otherwise remain hidden.

To further support our findings regarding alterations in intracluster distances and geometrical features of ellipses, we trained a linear encoder to classify mice based on either intracluster distance metrics or the configuration of the ellipses. Classifier performance—evaluated through accuracy, precision, recall, and F1-score—demonstrated significant discriminatory ability between WT+veh and 5xFAD+veh mice, as well as between 5xFAD+veh and 5xFAD+treat groups. In contrast, the encoder failed to distinguish between WT+veh and 5xFAD+treat mice, indicating highly similar manifold architectures in these groups. Demonstrated findings further support the beneficial effects of the treatment on hippocampal neuronal ensemble functioning, in agreement with conventional quantitative analyses [90]. To probe whether these manifold-based representations genuinely reflect physiological network dynamics, we shuffled the neuronal activity, thereby eliminating naturally occurring correlations between neuronal firings. As expected, in all comparisons—WT+veh vs. 5xFAD+veh and 5xFAD+veh vs. 5xFAD+treat—the encoder’s discriminative power significantly decreased. This result reinforces the idea that manifold representations reliably capture essential features of neuronal circuit activity. Interestingly, the classifier’s performance in distinguishing WT+veh from 5xFAD+treat mice remained unchanged after shuffling, staying at chance level. This further supports the conclusion that the manifold structures in these two groups are indeed similar, highlighting the restorative effect of the treatment. Altogether, these results underscore the positive impact of NDC-9009 treatment [90,95] and validate the utility of neuronal manifold analysis as a powerful method for assessing the functional state of complex neuronal networks.

To sum up, presented in the current study, an AI-driven algorithm for mice tracking and quantification of behavior type with parallel neuronal manifold analysis applied to hippocampal neuron calcium activity emerged with reliable and stable results. This methodology provides a powerful and scalable tool for studying brain–behavior relationships and offers new avenues for understanding neural circuit function in health and disease.

## 4. Materials and Methods

### 4.1. Animals

5xFAD mice (Jackson Laboratory, Bar Harbor, ME, USA; strain # 034848) [32] with a C57BL6 background were used for behavior and in vivo neuronal activity recordings in the rounded arena. The breeding colonies were established and maintained in a vivarium with 4–6 mice per cage and a 12 h light/dark cycle in the Laboratory of Molecular Neurodegeneration of Peter the Great St. Petersburg Polytechnic University vivarium with a 12 h light/dark cycle with ad libitum access to food and water.

### 4.2. Mice Treatment

Mice groups stated in the manuscript as “5xFAD+treat” were administered with 10 mg/kg NDC-9009 [90] (in a 10:10:80 ratio of DMSO, NDC-9009, and saline) for 2 weeks before and each day during imaging sessions. Mice groups “WT+veh” and “5xFAD+veh” were given a vehicle (DMSO and sterile saline) at the same volumes and with the same protocol.

### 4.3. Neuronal Network Training for Mice Tracking and Behavioral Scoring

Round arena marking. In the experiment, the arena was divided into four zones, as shown in the (Figure S1). To address the issue of arena marking, a novel algorithm based on analytical computer vision has been developed. The implementation of this algorithm was carried out using a combination of Python v3.11 and OpenCV v4.10. On the first step, the image was converted to grayscale. Then, the region of interest (ROI) was highlighted in the center of the image and is 4 times smaller in size than the original image. Using the built-in OpenCV functions, all possible contours within the ROI have been identified and marked in green. Using the built-in functions of OpenCV, the boundary of the arena was marked, which is marked in pink. The center of this circle is indicated by a red dot. Further, the contour closest to the center of the found circle is defined as the center of the arena. A circle is drawn around the remaining contours (that are not the centers) within the ROI (indicated in red). The center of this circle is the center of the arena identified in the previous step. This constitutes the first zone of the arena, namely, the central zone. The radius of the inner zone (red) is subtracted from the outer zone (pink). The resulting difference (blue) is then divided into three equal segments. Then, an inner area, a middle area, and an outer area were marked. Consequently, the dimensions of all sectors are ascertained, enabling us to precisely categorize any given location on the field based on its proximity to the center.

Framework. The project was implemented in Python using the following libraries: Ultralytics YOLO-Pose-v8—for training detection and classification models, OpenCV—for image analysis and arena segmentation, NumPy (1.26.4), Pandas (2.2.2)—for numerical data processing, Matplotlib (3.9.0), Seaborn (0.13.2)—for data visualization, and SciPy (1.14.0)—for signal filtering and smoothing (Savitzky–Golay filter). The project can be executed either through Python scripts or interactively in Jupyter Notebook (1.0.0). All models were trained and run on an NVIDIA RTX GPU, providing high performance for video processing. Required dependencies can be installed from the provided requirements.txt.

Model Training. The model used for animal pose estimation was YOLO-Pose-v8-m, trained on the annotated dataset with labeled key points by the MARS system [12]. The training pipeline included standard preprocessing and augmentation steps, along with loss functions specific to key-point detection tasks. The dataset consisted of 500 annotated images: 375 images were used for training, 125 images were used for validation, 80 images contained only the empty arena to improve model robustness and 25 images were synthetically generated through augmentation techniques. For the behavioral classification task, an additional dataset of 48,229 composite frames was created from mouse behavior videos. Each composite image was constructed using 21 consecutive frames, combined across color channels: the green channel represented the average of the preceding 10 frames, the red channel—the current frame, and the blue channel—the average of the subsequent 10 frames.

Tracking. Tracking was performed by applying the YOLO-Pose model frame-by-frame, followed by Kalman filtering to ensure temporal consistency. To further stabilize behavioral predictions, a mode smoothing algorithm was applied over a 25-frame window, reducing noise and transient misclassifications.

Animal Pose Estimation The pose estimation model predicted seven key points on the mouse’s body. The accuracy was evaluated using the PCK@0.25 metric (percentage of correct key points), where a prediction is correct if it lies within 0.25·D, with D being the inter-ear distance. The average PCK@0.25 score achieved was 82%.

Accuracy Metrics. Model performance was evaluated using the following metrics: PCK@0.25—for pose estimation accuracy, F1-score—for behavior classification performance, confusion matrix—for error analysis, key-point distance histograms—for assessing spatial prediction accuracy, trajectory plots and heatmaps—for visualization of movement patterns. To improve the reliability of behavioral classification, post-processing included Kalman filtering and statistical stabilization via mode smoothing. This helped eliminate outliers and ensured the interpretability of behavior sequences over time.

Behavior Classification. The YOLO-classification-v11 model was trained on the composite image dataset to classify mouse behavior. Dataset for training was 48,229 annotated images. The final model classified three behavior types: running, sitting, and grooming, achieving an F1 score of F = 0.971.

### 4.4. Viral Constructs Delivery and GRIN-Lens Implantation

All procedures are described in detailed in [8]. In short, all surgeries were performed under 1.5–2.0% anesthesia. A volume of 1.15 µL of viral constructs (AAV5.Syn.GCaMP6f.WPRE.SV40 [53]) at the infusion rate of 0.1 µL with titer more than 1 × 10^13^ vg/mL were injected unilaterally into left hippocampus (AP −2.1mm; DV −1.45mm; ML +1.4 mm) of mice using stereotaxic (68001, RWD Life Science, Guangdong, Shenzhen, China). Temperature was maintained at 37 °C by a heated mat with temperature controller (69,002, RWD Life Science, China). After 3 weeks, as appropriate level of genetically encoded calcium-indicator GCaMP6f was achieved, mice underwent GRIN-lens implantation. Cortex tissues were removed by aspiration under supply of PBS until corpus Collosum became visible [96]. Then, GRIN-lens was deepened at 1.45 mm below medial skull edge of the hole and glued with a small drop of superglue. Further, to fix lens to the skull, light-cured dental cement (Dent-light flow, tdVladmiva, Belgorod, Russia) was applied. At the end of lens implantation 50 µL of atipam was injected intraperitoneal and 1 mg/kg of dexamethasone. After 4–6 weeks, baseplate was fixed with the best FOV, where neurons or clear vessels were visible. Imaging sessions starts after total mice recovery after baseplating in 2 weeks.

### 4.5. Hippocampal Neuronal Activity Recordings Under Freely Behaving Conditions

Mice aged 6.5 months old were allowed to habituate to miniscope for 5 min once a day for 2 days. Experiments of neuronal activity recording in the freely moving mice were performed in the rounded arena with diameter of 63 cm. Hippocampal neuron activity was recorded for 7 min under the same conditions for 4 days. After each recording ended, the chamber was sterilized with 70% ethanol. Recording mice behavior during freely moving in a rounded arena was filmed by Webcam Logitech (C270HD, Apples, Switzerland).

### 4.6. Processing of Miniscope Recordings

Miniscope data were obtained using Pomidaq (Portable Miniscope Data Acquisition) (v. 0.4.5) at 15 frames per second. The first and the last minute of each session were cut, so individual recordings were was 5 min long. To process the miniscope data, we used Minian, an open-source tool for miniscope data analysis [27]. Minian performs background fluctuation elimination, motion correction, and calcium signal extraction using the CNMF method. The following parameters were applied in Minian: “wnd_size” set to 1000, “method” set to “rolling,” “stp_size” set to 500, and “max_wnd” set to 15, with default CNMF parameters.

Minian outputs an array with calcium activity traces (Ca^2+^ fluorescence) and neuron location data from the recording.

### 4.7. Datasets

In the current manuscript, we utilized the same miniscope imaging dataset as in our previous study [90]. In that earlier work, we investigated the effects of the SERCA PAM NDC-9009 on hippocampal neuronal ensembles using canonical analysis methods relying on individual neuron activation properties. Here, we extend our analysis by applying the same behavioral dataset to train the YOLO-8 and YOLO-11 neural network models. Additionally, the miniscope imaging data were employed to constrain neuronal manifolds and to correlate these manifold representations with distinct behavioral states. This approach aims to further elucidate the impact of Alzheimer’s disease pathology on hippocampal neuronal circuitry through advanced neuronal networks application for behavior scoring and neuronal manifold analysis.

### 4.8. Neuronal Manifold Construction Based on the Neuronal Calcium Traces

For the construction of manifolds and subsequent analysis aimed at identifying features associated with Alzheimer’s disease (AD), transgenic mice of the 5xFAD strain (6.5 months old) with a genetic model of AD were employed. Wild-type littermates of the same age were used as control subjects. Neuronal activity data from hippocampal ensembles were acquired using miniature fluorescence microscopy imaging. The dataset consists of time-series recordings of fluorescence intensity, reflecting the dynamics of the genetically encoded calcium indicator GCaMP6f over time for individual hippocampal neurons.

### 4.9. Preprocessing and Temporal Aggregation of Neural Activity

Given the sparseness of calcium signals in individual hippocampal neurons, we developed a preprocessing strategy aimed at capturing short-term temporal dynamics of the neural population. Rather than treating each frame as a point of manifold, neuronal activity across successive time intervals as a single extended observation. Specifically, the activity of all neurons across a fixed temporal window of 30 consecutive frames (corresponding to a 2 s of recording) was concatenated into a single high-dimensional vector:

$$x_{t}=\left[f_{t}^{T}f_{t\;+\;1}^{T}{\ldots f}_{t\;+\;29}^{T}\right]\in R^{30\;\times \;N},$$

where $f_{i}\in R^{N}$ represents the vector of fluorescence values for all N neurons at timeframe *i*. Each resulting vector *x_t_* encodes the full temporal sequence of neural activity over the 30-frame interval. This “flattening” strategy preserves the temporal dynamics of the ensemble and enables downstream manifold learning algorithms to capture not only instantaneous population states but also their evolution across short behavioral or physiological epochs. The resulting manifold embeds temporally structured neural motifs as individual points, facilitating the identification of group-specific features in reduced space.

### 4.10. Methods for Dimensionality Reduction

t-SNE (t-Distributed Stochastic Neighbor Embedding). t-SNE is a nonlinear dimensionality reduction technique that models high-dimensional data points as three-dimensional coordinates, preserving local similarities while emphasizing global structure. Specifically, similar objects are represented as closely positioned points, whereas dissimilar objects are mapped farther from each other with high probability. The method employs a t-distribution to mitigate crowding effects in low-dimensional space. The analysis utilized the scikit-learn [97] implementation of t-SNE, with the following key features: Euclidean distance was used to compute pairwise similarities and the Barnes–Hut approximation was applied to accelerate gradient descent, significantly reducing computational complexity.

PCA (Principal Component Analysis). PCA is a linear transformation method that identifies an orthogonal basis of principal components (PCs), maximizing variance in the data. These PCs form a new feature space, effectively reducing dimensionality while retaining the most informative projections. The scikit-learn PCA implementation was employed, with the automatic mean subtraction.

ICA (Independent Component Analysis). ICA decomposes a multivariate signal into statistically independent, non-Gaussian components by maximizing mutual independence. The scikit-learn FastICA algorithm was used with negentropy approximation and automatic sphering and centering for enhancing convergence.

MDS (Multidimensional Scaling). MDS projects data into a lower-dimensional space while preserving pairwise distances (or dissimilarities) from the original high-dimensional space. The scikit-learn MDS implementation relied on the SMACOF (Scaling by Majorizing a Complicated Function) algorithm.

UMAP (Uniform Manifold Approximation and Projection). UMAP constructs a weighted graph from high-dimensional data, where edge weights reflect local distances. The algorithm then optimizes a low-dimensional embedding by preserving the topological structure of this graph. Implementation The UMAP-learn library’s implementation was applied, with the nearest-neighbor search approximation and accelerated computation for large datasets.

Quantification of Manifold Structure

To quantify the between-group discrimination, we computed intracluster distances within the reduced-dimensional space:

$$Intracluster\;distance\left(C_{k}\right)=\frac{1}{n_{k}\times (n_{k}-1)}\sum_{i-1}^{n_{k}}\sum_{j\neq i}^{n_{k}}d\left(x_{i},x_{j}\right),$$

where *C_k_*: cluster containing *n_k_* data points; *x_i_*, *x_j_*: points within cluster *C_k_*; *d*(*x_i_*,*x_j_*). This metric inversely correlates with cluster compactness, where lower values indicate tighter neural population representations.

### 4.11. Error Ellipse Estimation

To characterize group-specific variability during particular behavioral states (running, sitting and grooming), we fitted 95% confidence ellipses to points within each manifold subset. The ellipses were computed by estimating the covariance matrix Σ of points assigned to the behavioral state and deriving its eigenvalues λ_1_, λ_2_ (where λ_1_ ≥ λ_2_), which determine the orientation and axes of the ellipse.

$$\mathrm{Area}\;\mathrm{of}\;\mathrm{ellipse}:\;S=\pi \times \sqrt{\lambda_{1}}\times \sqrt{\lambda_{2}},$$

$$\mathrm{Eccentricity}:\;c=\sqrt{1-\frac{\lambda_{2}^{2}}{\lambda_{1}^{2}}}.$$

These geometric descriptors served as features for subsequent classification analyses.

### 4.12. Control Analysis with Phase-Randomized Data

Neural activity vectors were temporally shuffled. All manifold construction and classification analyses were then re-run on this dataset.

### 4.13. Statistics

The Shapiro–Wilk or Kolmogorov–Smirnov tests were used to check normality of distributions. Comparisons were made using Student’s *t*-test or the Mann–Whitney test for paired analyses, and ANOVA followed by Tukey’s test or the Brown–Forsythe and Welch–ANOVA tests following Games–Howell’s multiple comparisons post hoc test, or the Kruskal–Wallis test followed by Dunn’s test for multiple comparisons. Statistical significance was set at *p* < 0.05. All data are presented as the mean ± standard error of the mean if others are not mentioned.

## Figures and Tables

**Table 1:** Comparison of the trained networks, YOLO-Pose-v8 and DeepLabCut, on the same dataset.

Key Point/Neuronal Network Approach	YOLO-Pose-v8	DeepLabCut
Nose	81.72	3.9
Left ear	88.17	26.0
Right ear	88.17	2.4
Tail	86.02	9.6

**Table 2:** Performance of the intracluster distance classifier in differentiating experimental groups for 10 independent repeats. Student’s *t*-tests or Mann–Whitney tests were used to compare classifier results based on neuronal manifolds with the corresponding one based on temporally shuffled neural activity (ns: non-significant, *: *p* < 0.05, **: *p* < 0.01). All data are presented as the mean ± SEM.

Classifier	Accuracy	Precision	Recall	F1-Score
Encoder performance based on the neuronal manifolds				
WT+veh vs. 5xFAD+veh	0.750 ± 0.016 **	0.764 ± 0.017 **	0.834 ± 0.019 *	0.814 ± 0.017 **
5xFAD+veh vs. 5xFAD+treat	0.673 ± 0.014 **	0.701 ± 0.010 **	0.7265 ± 0.020 **	0.700 ± 0.015 **
WT+veh vs. 5xFAD+treat	0.446 ± 0.014 ^ns^	0.395 ± 0.016 ^ns^	0.436 ± 0.013 ^ns^	0.402 ± 0.014 ^ns^
Shuffled				
WT+veh vs. 5xFAD+veh	0.612 ± 0.038	0.608 ± 0.043	0.642 ± 0.047	0.598 ± 0.048
5xFAD+veh vs. 5xFAD+treat	0.570 ± 0.024	0.611 ± 0.024	0.613 ± 0.031	0.596 ± 0.026
WT+veh vs. 5xFAD+treat	0.479 ± 0.022	0.429 ± 0.024	0.493 ± 0.028	0.448 ± 0.025

**Table 3:** Performance of the classifier based on the ellipses geometric properties in differentiating experimental groups for 10 independent repeats. Student’s t-test or the Mann–Whitney test was used to compare classifier results based on neuronal manifolds with the corresponding one based on temporally shuffled neural activity (ns: non-significant, *: *p* < 0.05, **: *p* < 0.01, ***: *p* < 0.001, ****: *p* < 0.0001 if the classifier results based on original data significantly higher; ns: non-significant, ^#^: *p* < 0.05, ^##^: *p* < 0.01 if the classifier results based on original data significantly lower). All data are presented as the mean ± SEM.

Classifier	Accuracy	Precision	Recall	F1-Score
Encoder performance based on the neuronal manifolds (running epochs)				
WT+veh vs. 5xFAD+veh	0.684 ± 0.007 ****	0.704 ± 0.009 ****	0.683 ± 0.010 ***	0.673 ± 0.008 ****
5xFAD+veh vs. 5xFAD+treat	0.623 ± 0.012 ****	0.663 ± 0.015 ****	0.664 ± 0.011 ***	0.648 ± 0.011 ***
WT+veh vs. 5xFAD+treat	0.420 ± 0.010 ^##^	0.363 ± 0.013 ^##^	0.408 ± 0.018 ^#^	0.374 ± 0.014 ^#^
Shuffled				
WT+veh vs. 5xFAD+veh	0.494 ± 0.023	0.500 ± 0.028	0.511 ± 0.035	0.475 ± 0.031
5xFAD+veh vs. 5xFAD+treat	0.470 ± 0.024	0.500 ± 0.028	0.471 ± 0.044	0.466 ± 0.035
WT+veh vs. 5xFAD+treat	0.498 ± 0.020	0.447 ± 0.025	0.503 ± 0.035	0.458 ± 0.028
Encoder performance based on the neuronal manifolds (sitting epochs)				
WT+veh vs. 5xFAD+veh	0.743 ± 0.012 ****	0.758 ± 0.013 ****	0.769 ± 0.014 ***	0.743 ± 0.012 ****
5xFAD+veh vs. 5xFAD+treat	0.634 ± 0.009 **	0.666 ± 0.006 **	0.692 ± 0.015 *	0.663 ± 0.011 **
WT+veh vs. 5xFAD+treat	0.495 ± 0.010 ^ns^	0.450 ± 0.012 ^ns^	0.504 ± 0.008 ^ns^	0.461 ± 0.009 ^ns^
Shuffled				
WT+veh vs. 5xFAD+veh	0.539 ± 0.031	0.534 ± 0.033	0.576 ± 0.042	0.529 ± 0.038
5xFAD+veh vs. 5xFAD+treat	0.525 ± 0.029	0.553 ± 0.029	0.573 ± 0.042	0.549 ± 0.035
WT+veh vs. 5xFAD+treat	0.490 ± 0.023	0.433 ± 0.028	0.489 ± 0.041	0.445 ± 0.033
Encoder performance based on the neuronal manifolds (grooming epochs)				
WT+veh vs. 5xFAD+veh	0.666 ± 0.014 ***	0.678 ± 0.017 ***	0.675 ± 0.012 ***	0.658 ± 0.014 ***
5xFAD+veh vs. 5xFAD+treat	0.665 ± 0.013 **	0.692 ± 0.014 ****	0.702 ± 0.013 *	0.681 ± 0.012 **
WT+veh vs. 5xFAD+treat	0.467 ± 0.015 ^ns^	0.379 ± 0.026 ^ns^	0.339 ± 0.025 ^ns^	0.339 ± 0.024 ^ns^
Shuffled				
WT+veh vs. 5xFAD+veh	0.523 ± 0.026	0.528 ± 0.032	0.542 ± 0.028	0.502 ± 0.026
5xFAD+veh vs. 5xFAD+treat	0.513 ± 0.029	0.534 ± 0.025	0.648 ± 0.036	0.575 ± 0.030
WT+veh vs. 5xFAD+treat	0.502 ± 0.026	0.425 ± 0.040	0.385 ± 0.041	0.384 ± 0.038

## Data Availability

The pretrained neuronal networks are deposited in an online repository, accessible by the following link: https://github.com/Biomed-imaging-lab/AI-mouse-detector (accessed on 15 July 2025) for an AI-based approach for mice position tracking and behavioral scoring, https://github.com/Biomed-imaging-lab/Neuro-manifold (accessed on 15 July 2025) for neuronal manifold construction based on calcium data. All the data are available upon reasonable request to corresponding authors.

## Supplementary Materials

The following supporting information can be downloaded at: https://www.mdpi.com/article/10.3390/ijms26157180/s1.
