# Supplementary material for: AI-Powered Mice Behavior Tracking and Its Application for Neuronal Manifold Analysis Based on Hippocampal Ensemble Activity in an Alzheimer’s Disease Mice Model
Source: Int J Mol Sci. 2025 Jul 25;26(15):7180. doi: 10.3390/ijms26157180 (PMC12346443; doi:10.3390/ijms26157180)
Supplement: Supplementary file 1 [file ijms-26-07180-s001.zip › Supplementary materials.pdf]

Supplementary Figures

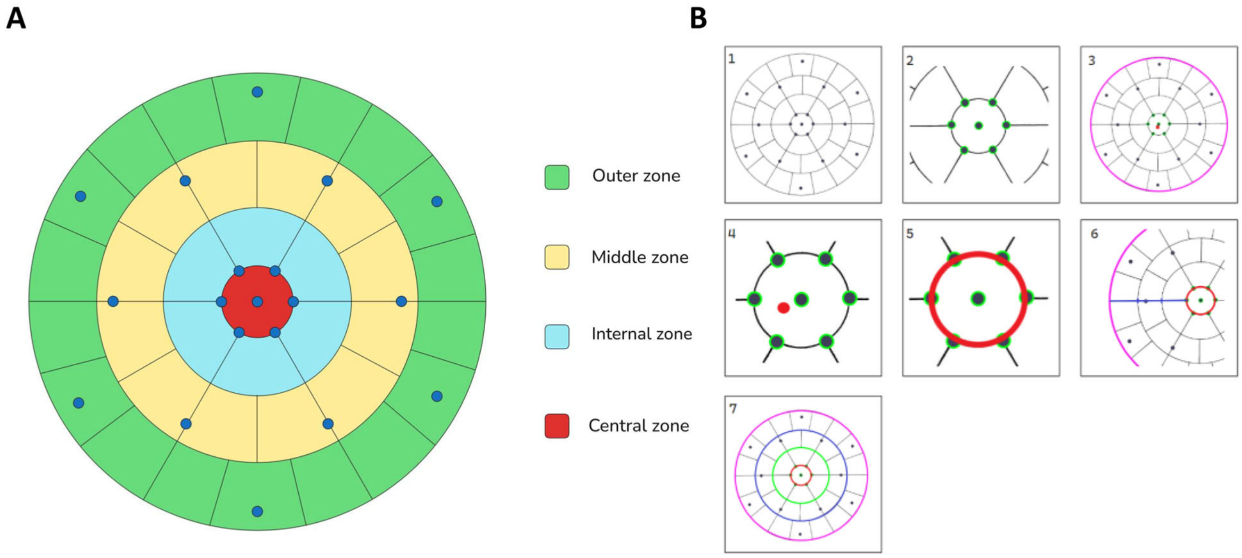

Supplementary Figure S1. Automatic annotation of rounded arena zones by computer vision algorithm. **(A)** Zones estimation in the rounded arena. **(B)** Algorithm for automatic zones marking using computer vision.

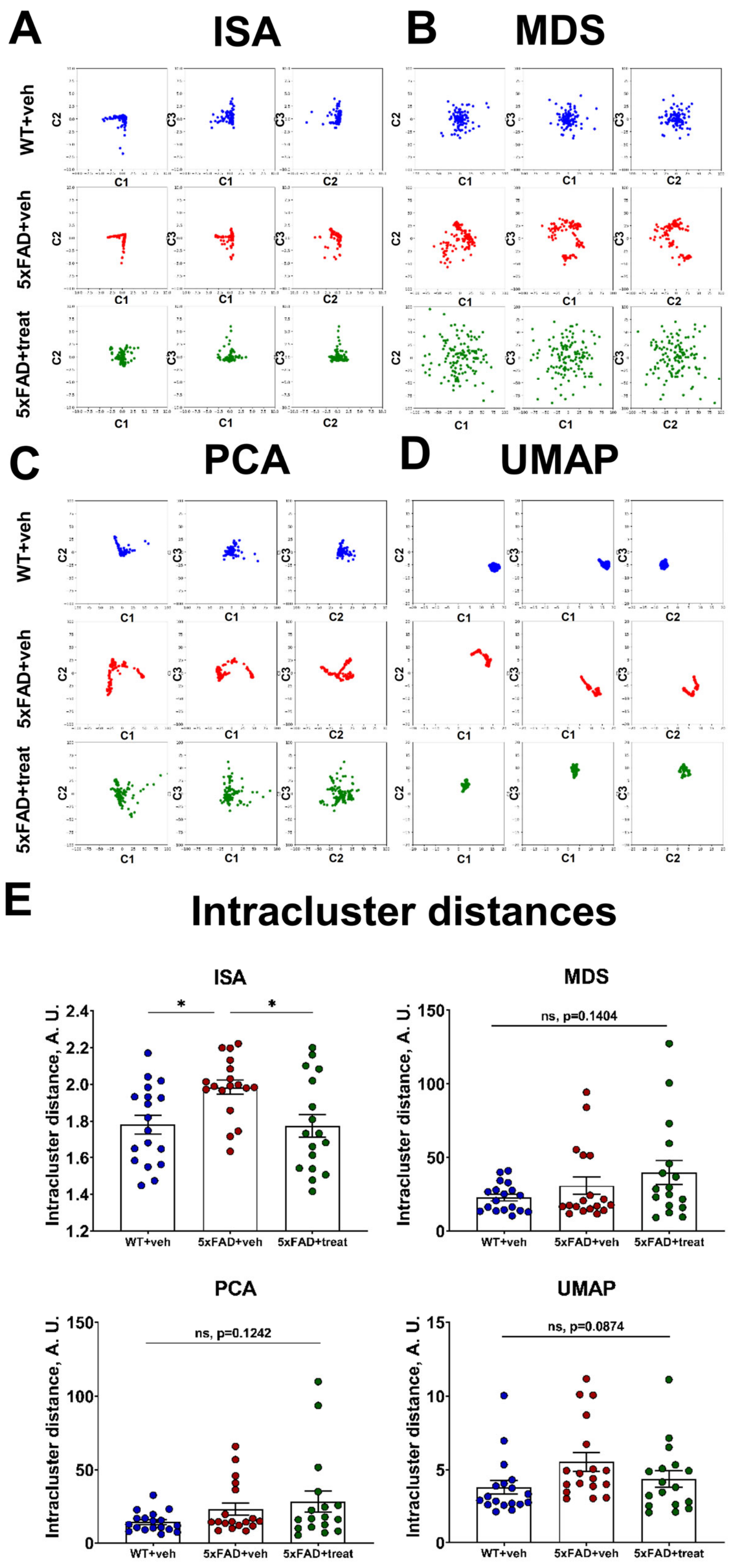

Supplementary Figure S2. Tested methods of dimensionality reduction lacking discriminative power. **(A), (B), (C)** and **(D)** Two-dimension representation of the neuronal manifold architecture for WT+veh, 5xFAD+veh and 5xFAD+treat mice groups using ISA, MDS, PCA and UMAP respectively. **(E)** Absence or deficiency in the discriminative power of ISA, MDS, PCA and UMAP between mice groups based on the intracluster distances.
